# Integrated analysis of high-throughput sequencing data shows abscisic acid-responsive genes and miRNAs in strawberry receptacle fruit ripening

**DOI:** 10.1038/s41438-018-0100-8

**Published:** 2019-02-01

**Authors:** Dongdong Li, Wangshu Mou, Rui Xia, Li Li, Christopher Zawora, Tiejin Ying, Linchun Mao, Zhongchi Liu, Zisheng Luo

**Affiliations:** 1 College of Biosystems Engineering and Food Science, Key Laboratory of Agro-Products Postharvest Handling Ministry of Agriculture and Rural Affairs, Zhejiang Key Laboratory for Agri-Food Processing, Zhejiang University, 310058 Hangzhou, P.R. China; 2 Department of Cell Biology and Molecular Genetics, University of Maryland, College Park, MD 20742 USA; 3 State Key Laboratory for Conservation and Utilization of Subtropical Agro-Bioresources, South China Agricultural University, 510642 Guangzhou, P.R. China

**Keywords:** Next-generation sequencing, Transcriptomics, Gene regulation

## Abstract

The perception and signal transduction of the plant hormone abscisic acid (ABA) are crucial for strawberry fruit ripening, but the underlying mechanism of how ABA regulates ripening-related genes has not been well understood. By employing high-throughput sequencing technology, we comprehensively analyzed transcriptomic and miRNA expression profiles simultaneously in ABA- and nordihydroguaiaretic acid (NDGA, an ABA biosynthesis blocker)-treated strawberry fruits with temporal resolution. The results revealed that ABA regulated many genes in different pathways, including hormone signal transduction and the biosynthesis of secondary metabolites. Transcription factor genes belonging to WRKY and heat shock factor (HSF) families might play key roles in regulating the expression of ABA inducible genes, whereas the KNOTTED1-like homeobox protein and Squamosa Promoter-Binding-like protein 18 might be responsible for ABA-downregulated genes. Additionally, 20 known and six novel differentially expressed miRNAs might be important regulators that assist ABA in regulating target genes that are involved in versatile physiological processes, such as hormone balance regulation, pigments formation and cell wall degradation. Furthermore, degradome analysis showed that one novel miRNA, Fa_novel6, could degrade its target gene *HERCULES1*, which likely contributed to fruit size determination during strawberry ripening. These results expanded our understanding of how ABA drives the strawberry fruit ripening process as well as the role of miRNAs in this process.

## Crop genetics: pathways to ripe strawberries

Researchers in China have uncovered the genetic factors through which the plant hormone ABA controls strawberry ripening. Zisheng Luo’s team at Zhejiang University used high-throughput sequencing to compare gene expression in strawberry plants after they were treated with ABA or an ABA-blocker. They discovered that ABA changes the expression of genes related to other hormones, metabolite synthesis, and breaking down cell walls. The team also checked the expression of short RNA molecules which regulate gene expression, known as microRNAs (miRNAs). They found 26 miRNAs which changed expression in response to ABA, six of which were novel, and identified 18 genes regulated by these miRNAs, including a cell wall gene which may be involved in fruit enlargement. These findings clarify the molecular mechanisms linking ABA with strawberry ripening and lay the groundwork for detailed functional studies.

## Introduction

The strawberry fruit is a model of nonclimacteric fruit. The fleshy part of strawberry fruit develops from the receptacle, and the hundreds of dried-out ovaries dotted on the receptacle surface are the true fruits. Strawberry fruit ripening is chiefly under the regulation of abscisic acid (ABA)^1,2^. The core ABA signal transduction pathway emerges as a double negative regulatory module in which the PYR/PYLs/RCARs receptors change their conformation when encountering ABA and then physically bind to clade A protein phosphatases 2C (PP2Cs). The inactivation of PP2Cs subsequently releases sucrose-nonfermenting kinase 1-related protein kinase 2 (SnRK2s), which is phosphorylated to activate transcription factors in the ABA response^3,4^. A previous study showed that gene silencing of *FaPYR1* in garden strawberry (*Fragaria ananassa*) significantly disrupted coloration in strawberry fruits, which could not be rescued by exogenous ABA application^1^. By contrast, when an important PP2C, *ABI1*, was silenced, strawberry fruit ripening was promoted^5^. These results demonstrate the important role of ABA signal transduction in strawberry fruit ripening. Additionally, the potential of improving not only strawberry but also other nonclimacteric fruit ripening through manipulations of the ABA signaling pathway is revealed.

Transcription factors (TFs) are crucial regulators for the transcription of ABA-responsive genes. For example, ABA can activate the expression of *R2R3-MYB10*, which has a general regulatory effect on many genes in the anthocyanin biosynthetic pathway, thereby controlling the strawberry fruit-ripening process^6^. Recently, another study indicated that *FaGAMYB* from the garden strawberry was a vital component in the interaction between gibberellin and ABA in strawberry receptacle ripening and was proposed to act upstream of the ABA signaling^7^. Likewise, another R2R3-MYB TF, *FaEOBII* (EMISSION OF BENZENOID II), plays a regulatory role in the scent formation of ripe strawberry fruit^8^. Despite progress in elucidating the importance of ABA signaling in strawberry fruit ripening, our understanding of how ABA regulates the downstream genes responsible for different physiological characteristics remains limited.

Plant microRNAs (miRNAs) are endogenous noncoding small RNAs approximately 21 nucleotides in length with high complementarity to their target mRNAs. The interaction between miRNAs and their targets commonly results in downregulation of target gene transcripts^9^. In some cases, although mRNAs are actively transcribed by TFs, their translation can be posttranscriptionally repressed by miRNAs^10^. Studies suggest that both known miRNAs, which are conserved within plants, and novel miRNAs, which are specific to particular species, are important regulators for fruit ripening because of fruit-specific expression^11,12^. Both types of miRNAs may cleave their target mRNAs that are responsible for different biochemical-physiological changes during the fruit-ripening process^13,14^. Therefore, whether miRNAs are involved in the ABA signaling pathway for the regulation of nonclimacteric fruit ripening remains of interest to investigate.

In the present study, the transcriptome and miRNA expression from strawberry fruits treated with ABA and nordihydroguaiaretic acid (NDGA, an ABA biosynthesis blocker) was profiled at 0, 5, and 8 days posttreatment. Genes in hormone signal transduction and biosynthesis of secondary metabolites pathways that are regulated by ABA were characterized, in addition to the important TFs in the process. Additionally, degradome sequencing results indicated that one novel miRNA in *Fragaria ananassa* strawberry, defined as Fa_novel6, might play a regulatory role in fruit enlargement by cleaving its target gene *HERCULES1* (*HERK1*). The present study not only revealed the specific genes and pathways that might mediate the effect of ABA in strawberry fruit ripening but also identified miRNAs as candidate posttranscriptional regulators of ABA responsible genes.

## Results

### Effects of exogenous ABA and NDGA treatment on strawberry fruit ripening

**Fig. 1:**
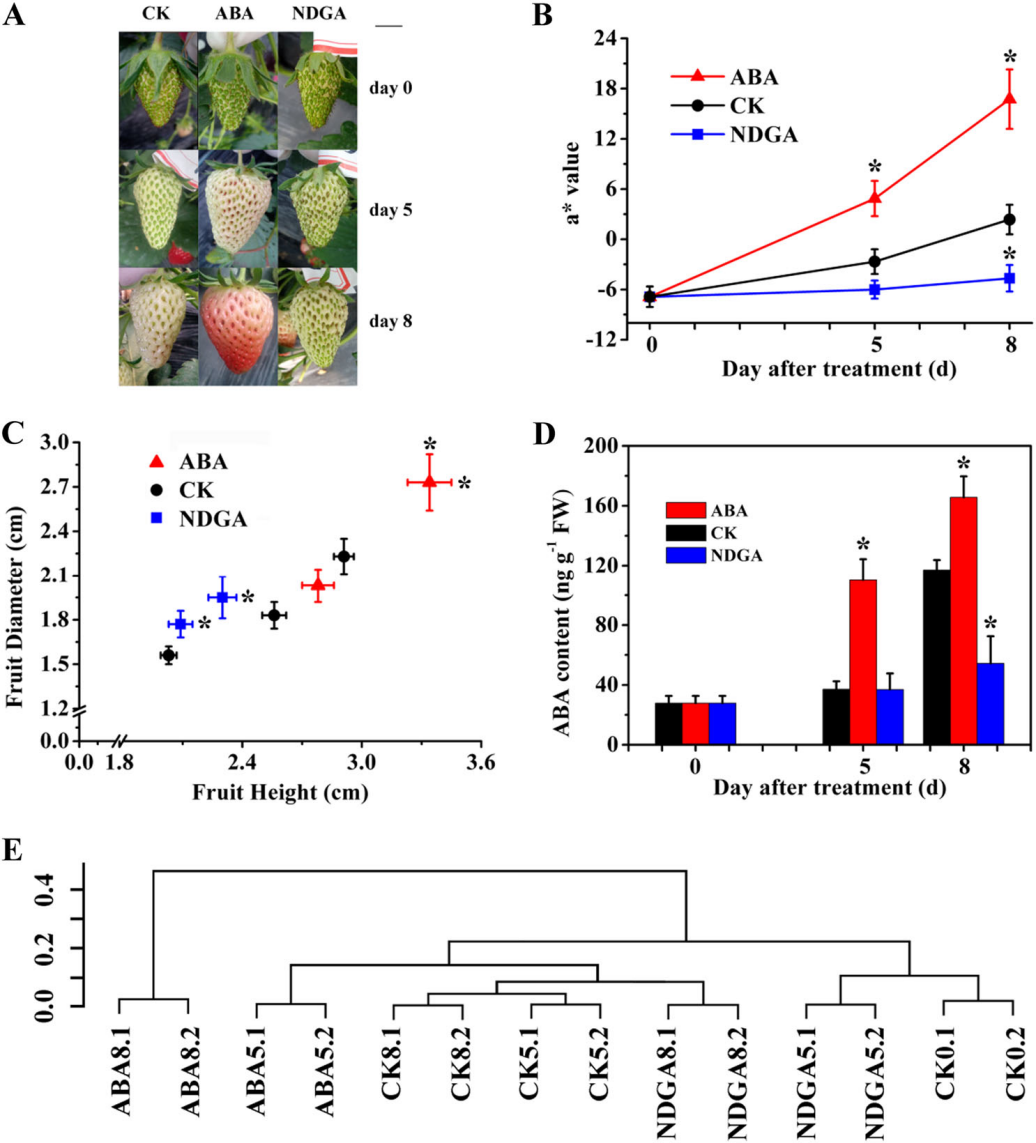
Effects of exogenous ABA and NDGA treatment on strawberry fruit ripening and cluster dendrogram of transcriptome samples. **a** Morphological changes in fruits at 0, 5, and 8 days posttreatment. Hormone treatment was applied to 2-week-old strawberry fruits after anthesis, and the day of treatment was set as day 0. CK represents the control. In all figures to follow, samples are named in a rule of treatment and sample collection day. **b** Changes in fruit color a* values in the L*a*b* color space. **c** Changes in diameter and height of fruit size. Significance is labeled with an asterisk on the right of the symbol in the height dimension and on the top for the diameter dimension. **d** Changes in ABA content. **e** Cluster dendrogram of the transcriptome sample replicates and among different treatments. *P < 0.05

Exogenous ABA treatment remarkably accelerated fruit ripening, while NDGA inhibited the process, as shown in Fig. 1a. The a* value (higher values represent redder in the L*a*b* color space) increased much faster in ABA-treated fruits than that in the control during ripening, and significantly higher levels were observed in ABA5 and ABA8 (Fig. 1b; ABA5 indicates fruits 5 days after treatment with ABA). However, NDGA treatment delayed the increase in fruit color (Fig. 1b). Moreover, results for fruit size showed that ABA-treated fruits grew slightly larger based on higher values of fruit diameter and height (Fig. 1c), whereas significantly lower levels of fruit height were observed in NDGA-treated fruits (Fig. 1c). Additionally, exogenous ABA treatment substantially increased ABA content in the fruits, whereas NDGA treatment resulted in reduced ABA accumulation 8 days after treatment (Fig. 1d). Our results indicated that ABA was a critical plant hormone for strawberry fruit ripening. A similar result of promoted fruit coloring by ABA treatment was also observed previously^2^. However, in another report, exogenous ABA treatment did not promote fruit ripening, although ABA content continually increased during fruit ripening^15^. This finding could be explained by different hormone treatment concentrations and treatment techniques.

### Transcriptional profiles of differentially expressed genes and KEGG pathway analysis

Sampling the receptacles of different treatments at different stages resulted in 14 transcriptomes (7 samples replicated by 2). The correlation dendrogram showed that all paired biological replicates were clustered together (Fig. 1e). The global relationships among the seven samples suggested effective regulatory influences of ABA and NDGA on fruit ripening. For example, ABA treatment at day 8 differed most from all other samples, which was also evident from the morphology of the fruits (Fig. 1a).

**Fig. 2:**
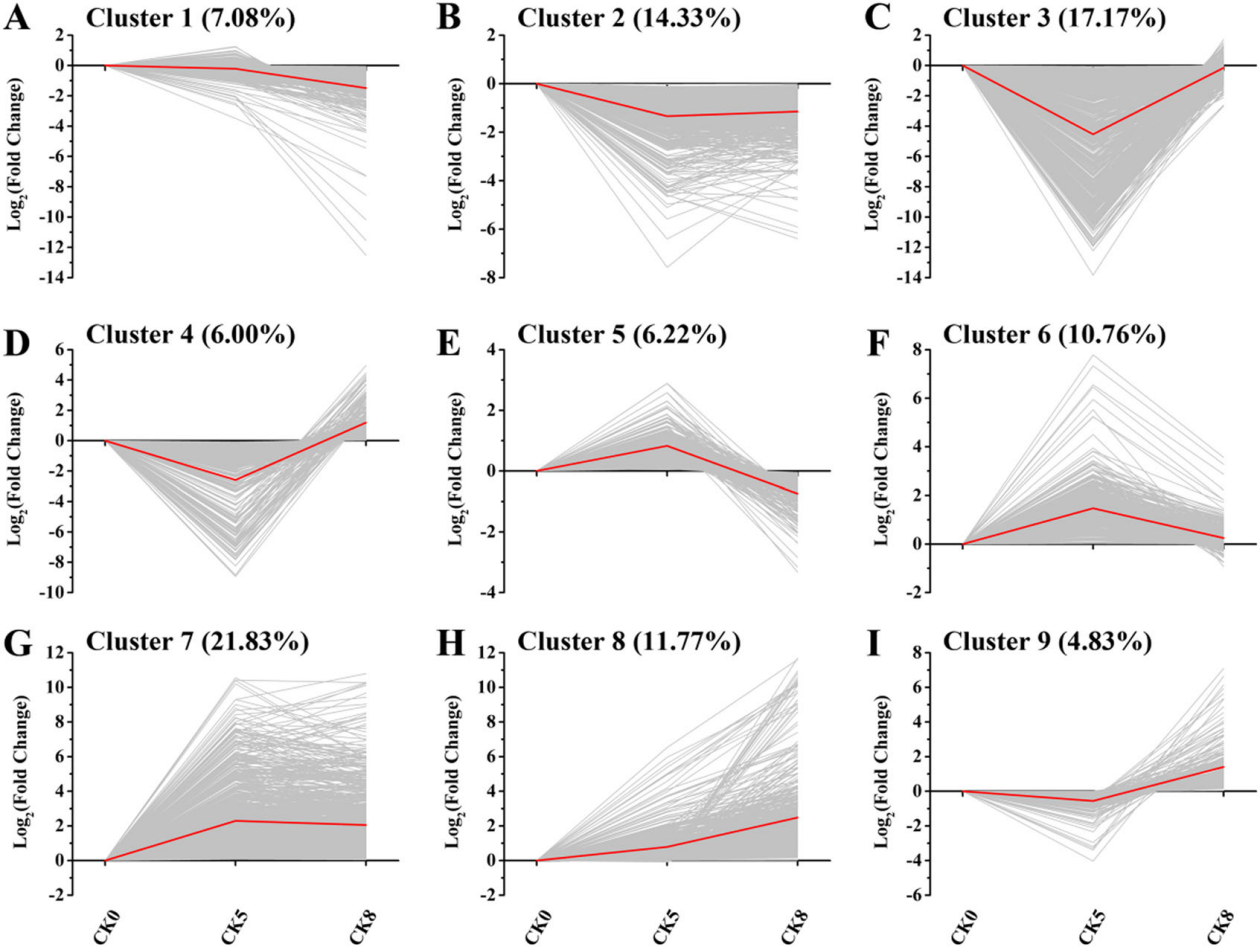
Nine clusters of the 4164 differentially expressed genes in ABA5 vs. CK5 and/or NDGA5 vs. CK5 and/or ABA8 vs. CK8 and/or NDGA8 vs. CK8. The red line indicates the global expression profile

A total of 167,564 unigenes were de novo assembled with annotations obtained both from *Fragaria vesca* (from PLAZA https://bioinformatics.psb.ugent.be/plaza/versions/plaza_v3_dicots/) and *Fragaria ananassa* (from the Strawberry GARDEN site http://strawberry-garden.kazusa.or.jp/; Supplemental Table S1). Expression of all these unigenes was compared using DESeq for the following: ABA5 vs. CK5, ABA8 vs. CK8, NDGA5 vs. CK5 and NDGA8 vs. CK8. Ultimately, 4164 genes were identified as differentially expressed genes at least in one comparison (Supplementary Table S2). To better understand the regulatory effects of ABA and NDGA on these genes, we first identified their expression profiles in control fruits across the three time points. Genes with similar expression trends were combined into one cluster. In the present study, nine clusters were created based on gene expression fold change compared with CK0 (Fig. 2). Clusters 1 and 2 showed that expression of approximately 21% of the 4164 differentially expressed genes declined as fruit ripened (Fig. 2a, b), such as PP2Cs (Supplementary Table S2). Previously, the expression level of an important PP2C, *ABI1*, declined rapidly during strawberry fruit development^5^. Furthermore, the expression of approximately 38% of the genes increased during fruit development as combined in clusters 7, 8, and 9 (Fig. 2g−i), including those of heat shock TFs (Supplementary Table S2), which play a critical role in stress responses^16^. For some genes, the expression was significantly up- or downregulated as illustrated in clusters 3 and 6, respectively (Fig. 2c, f). Notably, the trends of a minority of genes in clusters 4 and 5 were much more complicated, with remarkable fluctuations in expression levels (Fig. 2d, e).

The pathway analysis revealed that these 4164 differentially expressed genes were most enriched in the metabolic pathway (28.82%), biosynthesis of secondary metabolites (12.95%), and plant hormone signal transduction (5.76%), indicating a specific regulatory influence of ABA and NDGA on the expression of genes in these pathways (Supplemental Fig. S1). Thereafter, we focused attention on the pathways of biosynthesis of secondary metabolites and plant hormone signal transduction, which might help to elucidate the regulatory effects of ABA on ripening-related genes.

### Differentially expressed genes involved in hormone signal transduction and the biosynthesis of secondary metabolite pathways

**Fig. 3:**
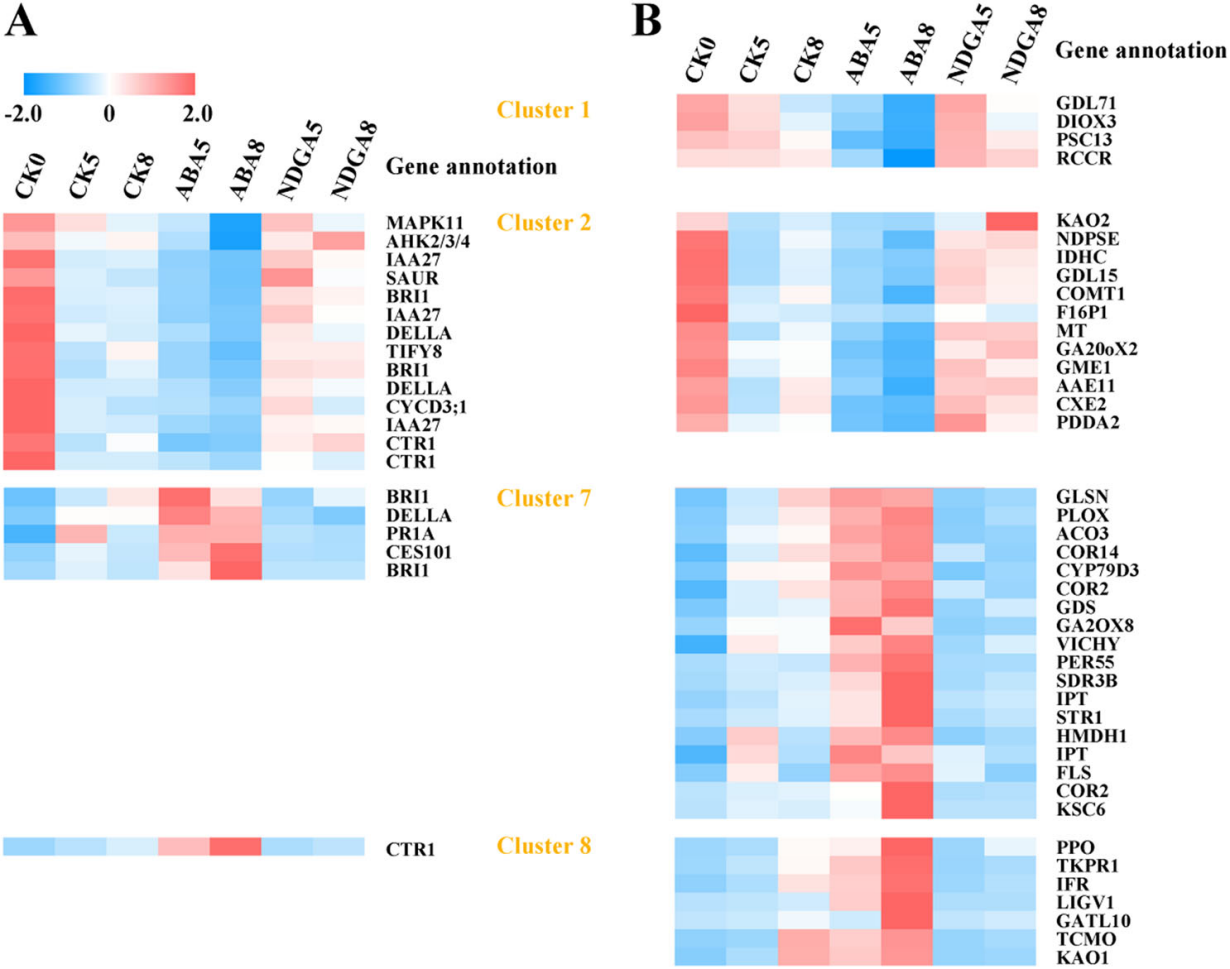
Expression of ABA specifically regulated genes involved in hormone signal transduction and biosynthesis of secondary metabolites pathways. **a** Genes involved in hormone signal transduction. **b** Genes involved in biosynthesis of secondary metabolites. The heat map relative value is transformed to a Z-score from the average RPKM and deviation of a gene among the samples. Red indicates relatively high expression; blue indicates relatively low expression. Clusters are referred to in Fig. 2. For full names of annotation, refer to the supplementary files

Of the 120 and 270 differentially expressed genes annotated in the pathways of hormonal signaling and biosynthesis of secondary metabolites, respectively, 20 hormonal and 41 secondary metabolite synthesis genes were regulated by ABA, respectively (Fig. 3 and Supplementary Table S3). In clusters 1 and 2, genes were expressed at relatively low levels in ABA-treated fruits, whereas relatively high levels were maintained in NDGA-treated fruits. Conversely, gene expression increased with ABA and was suppressed by NDGA in clusters 7 and 8. In the hormone signaling transduction pathways, the cytokinin receptor gene *AHK2/3/4* was downregulated by ABA. In this signaling pathway, one important downstream component *CYCD3*, which is pivotal for cell division, was downregulated (Fig. 3a). As for brassinosteroids, one receptor *BRI1* unigene was downregulated, whereas two other *BRI1* unigenes were upregulated (Fig. 3a). Regarding auxin, *IAA27* and *SAUR* genes were downregulated in ABA-treated fruits. Both DELLA and TIFY8 are well-known transcriptional repressors in hormonal signaling pathways^17,18^. The results showed that in the gibberellin pathway two *DELLA* unigenes in cluster 2 were downregulated, whereas another *DELLA* unigene in cluster 7 was upregulated by ABA. With regard to jasmonic acid and salicylic acid, the *TIFY8* gene and the *PR1* gene were respectively down- and upregulated by ABA. Moreover, genes in the ethylene signaling pathway, including *MAPK11* and *CTR1*, were also regulated by ABA (Fig. 3a). Notably, ABA signal transduction-related genes were not identified as ABA-regulated. These results indicated that ABA might regulate other hormonal signal transduction pathways during strawberry fruit ripening, such as the inhibition of auxin.

ABA prompted a decrease in the expression of *KAO2* and *GA20oX2*, which are key genes for gibberellic acid biosynthesis, in cluster 2 (Fig. 3b). The expression of *ACO3* in ethylene biosynthesis was upregulated by ABA. Additionally, genes such as *RCCR* involved in chlorophyll degradation were downregulated; whereas the *PPO* gene was upregulated in ABA-treated fruits. Likewise, *galacturonosyltransferase-like 10* (*GATL10*), which might be involved in cell wall biosynthesis^19^, was also upregulated by ABA (Fig. 3b). These results showed the versatile regulatory effects of ABA on secondary metabolites during fruit ripening.

### ABA activated or suppressed TFs, which might act as pivotal regulators for gene expression

**Fig. 4:**
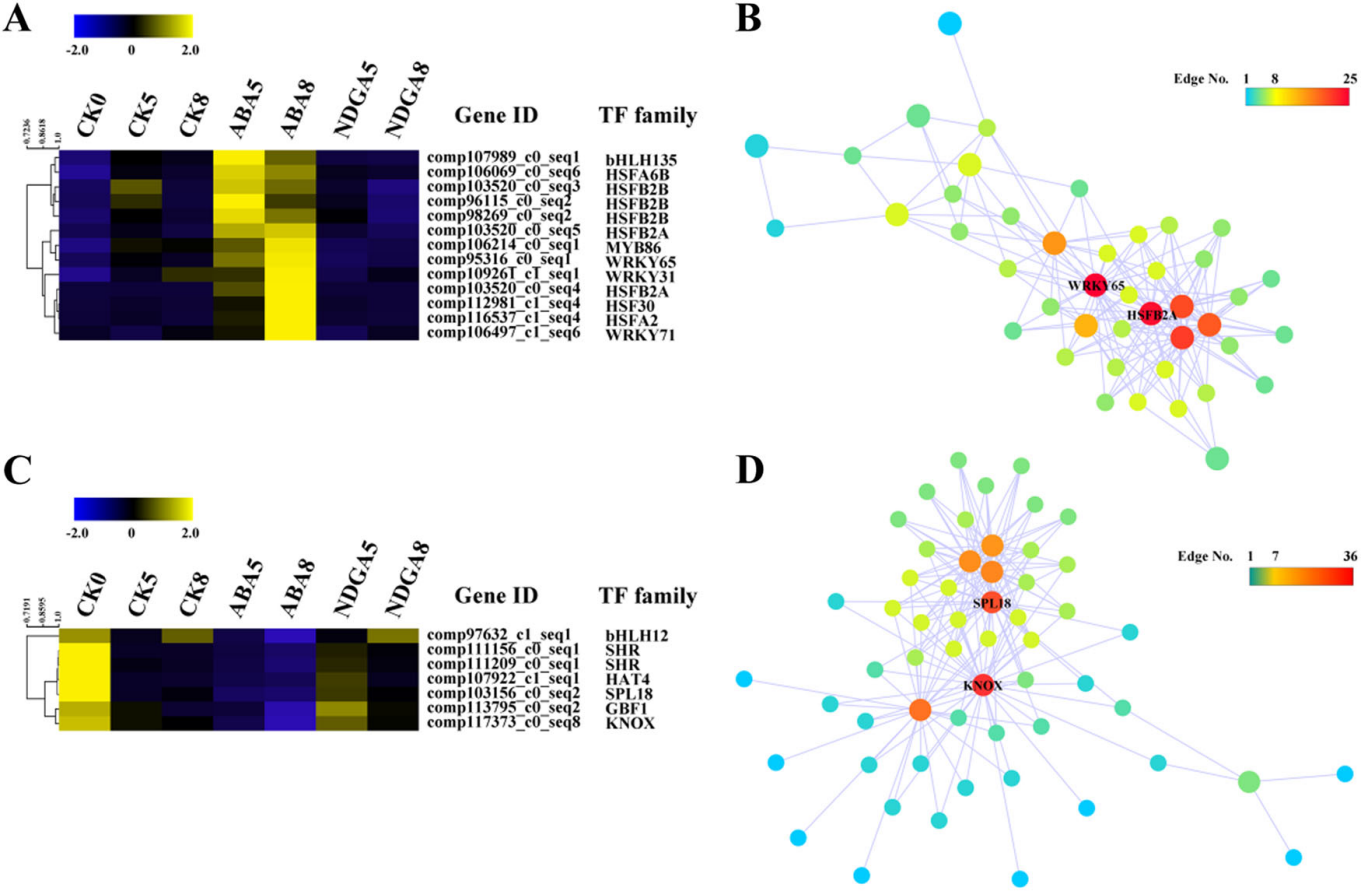
Transcription factors specifically regulated by ABA. **a** Heat map shows 13 transcription factors specifically upregulated by ABA. **b** The correlation network of the 13 upregulated transcription factors and 30 genes. **c** Heat map shows seven transcription factors specifically downregulated by ABA. **d** The correlation network of the seven upregulated transcription factors and 89 genes. The cutoff value for edge connecting is 0.9. Transcription factors are shown by larger circles

TFs are crucial for the regulation of gene expression. In our study, 110 differentially expressed genes were annotated as TFs belonging to 36 families (Supplementary Table S4). Among these genes, compared with expression in the control, 13 TFs were expressed higher in ABA-treated fruits and lower in NDGA-treated fruits (Fig. 4a). The most abundant TF family was the HSF family, with eight genes identified. Moreover, genes of TF families such as WRKY, MYB and bHLH were also promoted by ABA (Fig. 4a). By contrast, the expression of seven TFs were downregulated in ABA-treated fruits, whereas the expression was retained in NDGA (Fig. 4c). Different *bHLH* genes were regulated in opposite directions (Fig. 4a, c), which was not surprising given the diverse functions of *bHLH* genes.

We further constructed two gene expression networks to elucidate the possible roles these TFs played in fruit ripening (Fig. 4b, d). The network illustrated the expression correlations between transcription factors and other genes. In the first network, 30 genes with a threefold increase in expression with ABA and a twofold decrease with NDGA were used (Supplementary Table S4). Genes including those encoding UDP-glycosyltransferase and long-chain-alcohol O-fatty-acyltransferase were identified. Based on the coexpression correlation rates (>0.9) between the TFs and these 30 genes, a total of 162 edges were created. The hub TFs HSFB2A and WRKY65 had the top two highest edge numbers (Fig. 4b), highlighting them as candidate key regulators for the ABA-induced fruit ripening process. In the second network, seven TFs were downregulated by ABA, whereas these TFs were upregulated with NDGA. Consistent with the expression profiling of these TFs, 89 ABA-downregulated and NDGA-upregulated genes were used. Genes including those encoding chlorophyll synthase and cytokinin dehydrogenase were annotated among the 89 genes. In this network, knotted1-like homeobox (KNOX) belonging to the TALE family and Squamosa promoter-binding-like protein 18 (SPL18) belonging to the SBP family were the hub TFs with the highest edge numbers. Collectively, these results identified candidate TFs that might act to mediate the effect of ABA.

### Differentially expressed known and novel miRNAs

**Fig. 5:**
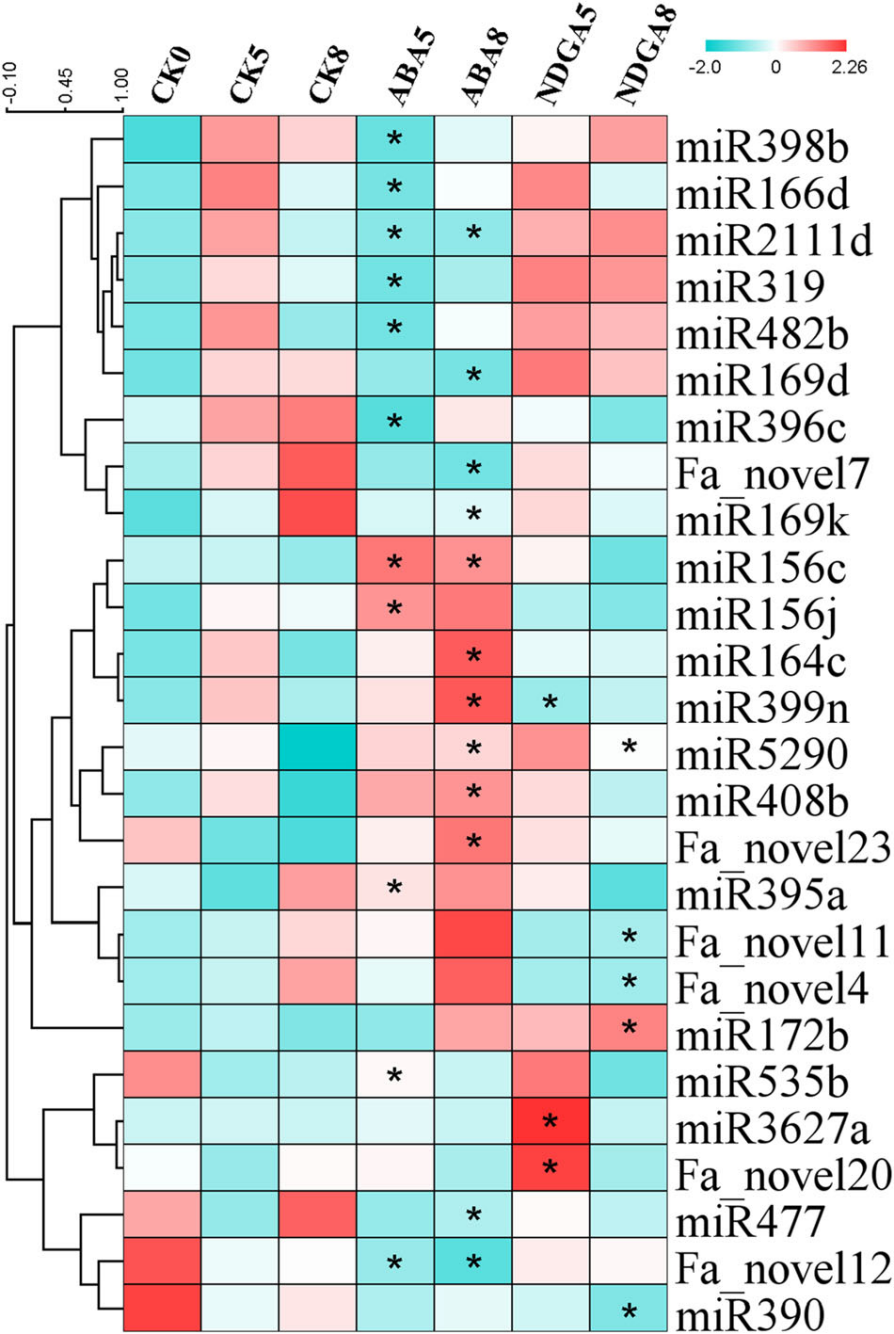
Differentially expressed known and novel miRNAs. Blue indicates relatively low expression; red indicates relatively high expression. *Indicates a statistically significant change in miRNA expression

MiRNAs play various roles in fruit development and ripening, targeting genes that are at crucial nodes of regulatory networks^9,11,20^. Therefore, we constructed 14 small RNA-sequencing libraries (7 distinct samples × 2 replicates) mirroring the previous RNA-seq to investigate the roles of miRNAs during fruit ripening. The sequencing depth was at least 13,852,941 raw reads that led to 12,678,630 clean reads after removing the low-quality reads (Supplementary Table S5). After mapping to miRbase 21.0 (http://www.mirbase.org/), we identified a total of 47 known miRNAs belonging to 25 miRNA families and 28 novel miRNAs in all fruit samples (Supplementary Table S6). Among these miRNAs, 20 known miRNAs and 6 novel miRNAs were differentially expressed in at least one ABA or NDGA sample (Fig. 5). Known miRNAs such as miR398b and miR166d were downregulated in ABA5 compared with those in CK5. One known miRNA, miR2111d, was continuously downregulated in ABA-treated fruits, whereas miRNAs such as miR156c were upregulated both in ABA5 and ABA8 (Fig. 5). In NDGA8, miR172b was upregulated. Regarding the novel miRNAs, we found Fa_novel7 was downregulated in ABA-treated fruits. As computationally predicted, the target gene of Fa_novel7 was *sugar transporter ERD6-like 3*. By contrast, Fa_novel23, with the target *Kelch domain-containing F-box protein* (*KFB*), increased expression in ABA8 fruits. In NDGA-treated fruits, Fa_novel11 and Fa_novel4 were downregulated, whereas Fa_novel20 was upregulated. Notably, the target of Fa_novel11 was predicted to be a pectin methylesterase inhibitor (*PMEI*) gene, which encodes a vital inhibitor for pectin methylesterases. Therefore, Fa_novel11 might regulate fruit cell wall degradation and softening. These results revealed the influences of ABA both on known and novel miRNA expression, indicating miRNAs might act together with ABA to regulate genes in the fruit-ripening process.

### Degradome analysis of differentially expressed miRNAs and target mRNAs

**Fig. 6:**
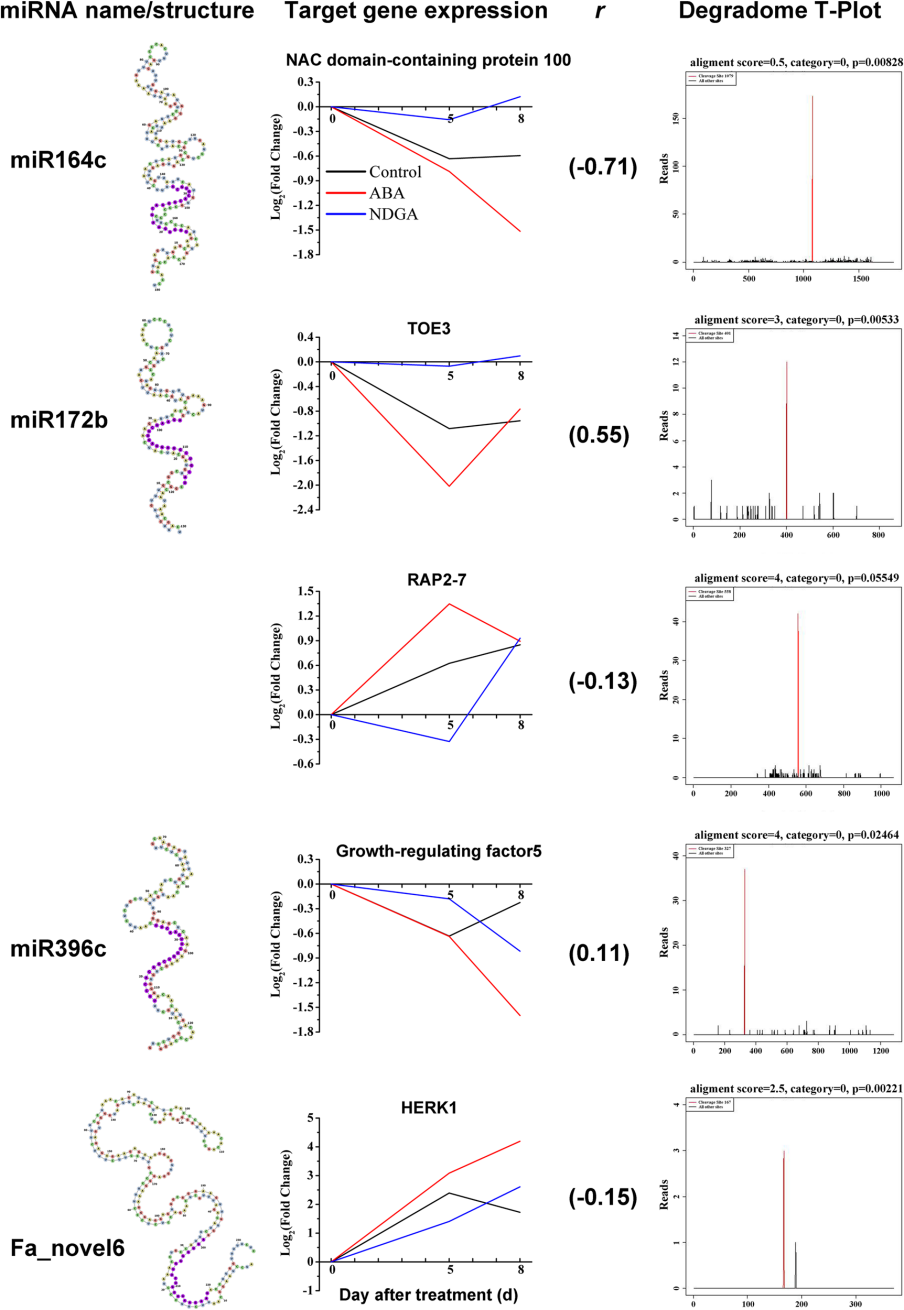
Degradome sequencing validated that three known miRNAs and one novel miRNA degraded their corresponding target genes. The secondary structure, expression of target gene, correlation rate between miRNA and target gene, and degradome T-plot of miR164c, miR172b, miR396c and Fa_novel6

MiRNAs function predominantly by pairing with their target mRNAs and causing mRNA degradation. Degradome sequencing is basically a high-throughput method of identifying cleavage products of mRNAs mediated by miRNAs^14,21^. We performed degradome sequencing to identify strawberry miRNA targets. A total of 175,880,715 raw reads were obtained from the sequencing, and at least 43,748 transcripts were covered (Supplemental Table S7). We found a total of 18 target genes that were degraded by different miRNAs (Supplemental Table S7). Among the miRNAs, three known differentially expressed miRNAs, miR164c, miR172b, and miR396c (Fig. 5), were validated to degrade their corresponding target genes, *NAC domain-containing protein 100*, *TARGET OF EAT 3* (*TOE3*) and *RAP2-7*, *growth-regulating factor5*, respectively (Fig. 6). Importantly, the target gene of Fa_novel6 was *HERK1*, a gene that previously was shown to participate in the regulation of cell elongation during vegetative growth^22^. MiRNAs usually negatively regulate their targets by degradation or posttranscriptional inhibition; however, a high negative correlation rate in gene expression between a miRNA and its target was only observed for the miR164c to *NAC domain-containing protein 100* pair. We speculate that some other regulatory mechanisms might be involved for the miRNAs. The degradome T-plot showed a clear single peak at the degradation site, which was between the 10th and 11th nucleotides pairing the miRNA and its target (Fig. 6). The highlighted red peak in the plot shows the reads abundance of a 3′-cleaved target fragment, demonstrating that many target transcripts were enriched in the sequenced degradome data at the miRNA targeting position. We applied the most stringent criteria that the expected miRNA cleavage site had the most abundant reads matching the target transcript (Fig. 6). Although these three known miRNAs were differentially expressed, their target genes were not found in any of the differentially expressed unigene clusters. These results indicated that target gene expression might also be modulated by other regulators or compensated under feedback regulation. By contrast, Fa_novel6 was not significantly differentially expressed, but its target, *HERK1*, was found in cluster 7, which showed increased expression as the fruit ripened (Fig. 2).

## Discussion

In the present work, applications of ABA and NDGA to the strawberry receptacle were used to identify ABA-regulated genes, which provided new insights into the molecular mechanisms of ABA regulation of strawberry fruit ripening. Unlike many high-throughput sequencing studies, our strategy of pairwise comparisons between ABA- or NDGA-treated fruits and controls allowed us to identify genes regulated by ABA in many pathways. For example, ABA likely interacts antagonistically with salicylic acid upon plant pathogen infection^23,24^, whereas other studies report that ABA synergistically cooperates with salicylic acid to increase abiotic stress tolerance^25,26^. Our results showed that the very downstream gene *PR1* in the salicylic acid signaling pathway was upregulated in response to ABA (Fig. 3a), extending our understanding of the interaction between ABA and salicylic acid signaling during fruit ripening. However, this strategy might miss genes whose expression may be subjected to feedback mechanisms. For example, NDGA is an effective inhibitor for the key ABA biosynthetic enzyme 9-*cis*-epoxycarotenoid dioxygenase (NCED). A negative feedback loop could increase *NCED* expression in response to NDGA. Simultaneously, the *NCED* gene was also induced by ABA; thus, this gene may buffer gene expression differences. Another example found in our data was *CYP707A1* (comp94002_c0_seq1 in Supplemental Table S1), which encodes the ABA hydroxylase for ABA degradation and was downregulated in both ABA- and NDGA-treated fruits. Expression of *FaCYP707A1* decreased as fruit ripened^27^, whereas ABA content accumulated rapidly at the late stage during fruit ripening^2^. Therefore, the ABA treatment causing a decrease in the expression of *CYP707A1* was reasonable. By contrast, NDGA blocked ABA biosynthesis and resulted in reduced expression of *CYP707A1* since the fruits require additional ABA with inhibition of the degradation pathway of ABA homeostasis. Moreover, a similar situation was also observed for *PYR1* (comp99969_c0_seq2 in Supplemental Table S1), a vital receptor gene in ABA signaling. *PYR1* expression increased gradually as fruit developed and peaked in light green fruit^1^. In our results, ABA treatment might result in the decreased expression of *PYR1* during late stage of fruit ripening; whereas NDGA inhibited its expression. In this case, *PYR1* was not observed as a differentially expressed gene responsive to ABA. Moreover, note that the transcript profiles of differentially expressed genes were clustered based on the expression in control fruits, which did not cover the entire ripening stage; therefore, our ability to analyze genes with much more complicated expression, such as genes in clusters 4 and 5, was restrained (Fig. 2). Nevertheless, our results can contribute to elucidating the molecular mechanism of how ABA drives fruit ripening, because the transition from the large green stage to the white stage was a critical period when the endogenous ABA content increased dramatically^2^.

Previous research shows that the achene and receptacle are distinct from one another. For example, the expression of two genes encoding 1-aminocyclopropane-1-carboxylic acid (ACC) synthase and ACC oxidase, which are critical for ethylene biosynthesis, was stage and organ specific^28^. In the receptacle, *FaGAMYB* activates the ripening program by stimulating ABA and sucrose signaling^7^. Kang et al.^29^ used RNA-seq to profile five different early stages of *Fragaria vesca* fruit in finely dissected tissues and uncovered distinct transcriptomes between the receptacle and achene. More recently, differences in the ripening program between the achene and receptacle in garden strawberry were also revealed by RNA-seq^30^. Whereas the above studies used RNA-seq to study different tissues and stages during fruit development, the current study provided transcriptome information of the receptacle that was treated with ABA to uncover genes acting downstream of ABA. Functional studies with transgenic plants will be required to further investigate specific molecular mechanisms of how ABA regulates the ripening program in both the strawberry receptacle and achene.

Fruit development and ripening are under symphonic regulation of phytohormones at each phase^31^. Previously, studies demonstrated that auxin played a much more important role in the early stage of strawberry fruit development^15,29^. Here, we showed that *IAA27* was downregulated by exogenous ABA treatment (Fig. 3). In Arabidopsis, Aux/IAAs interact with ARFs, creating various functional effects for auxin signaling^32^. Therefore, the downregulation of *IAA27* might be a consequence of less ubiquitination degradation or requirement for ARF repressor release^32^. Previously, a protein−protein interaction map in Arabidopsis showed that IAA27 interacts with many ARFs, such as ARF8, with a strong relation^33^. ARF8 can function as an important regulator in flower coloring by stimulating expression of *anthocyanin synthase* and *dihydroflavonol reductase* genes^34^. In our study, although no significantly differentially expressed ARF genes were identified, the downregulated expression of *IAA27* by ABA might cause some comprehensive effects on many members in the ARF family. Although classified as a nonclimacteric fruit, strawberry fruits produce ethylene and express ethylene receptors genes^35,36^. Aharoni and O’connell^37^ proposed that ethylene might play an important role in achene maturation. Transgenic strawberry plants overexpressing *etr1-1* with diminished sensitivity to ethylene not only exhibit altered achene color and weight but also altered metabolites including amino acids, sugars and organic acids both in the achenes and receptacle^28^. Our results showed that the ethylene biosynthesis and signaling genes *ACO3* (comp69739_c0_seq1) and *CTR1* (comp102922_c0_seq7) were upregulated by ABA, suggesting cross talk between ABA and ethylene during strawberry fruit ripening. For cytokinin, the receptor gene *AHK2/3/4* was downregulated by ABA, suggesting that cell division was attenuated before cell expansion. Similarly, *CYCD3*, which is critical for brassinosteroid-regulated cell division, was also downregulated by ABA (Fig. 3a). ABA-promoted fruit enlargement might occur via increasing the transcript abundance of *GATL10*, which was previously determined to be involved in cell wall biosynthesis in Arabidopsis^19^.

The MYB, bHLH, and WD-repeat proteins form a transcriptional complex with pivotal roles in regulating anthocyanin biosynthesis^38^. *FaMYB10*, a member of the complex, is the most well-studied transcription factor that regulates the biosynthesis of the red pigment in strawberry fruits. The expression was stimulated by ABA^6^. RNAi downregulation of *FaMYB10* causes drastic reduction in anthocyanin content^6^. By contrast, overexpression of *FaMYB10* elevates anthocyanin levels not only in the fruit but also in other plant tissues^39^. Further, a single nonsynonymous SNP that causes amino acid W to change to S in *FveMYB10* is responsible for the pale yellow color in different diploid strawberry accessions^40^. Previous studies indicate that *FaMYB1* is a negative regulator of anthocyanin biosynthesis in strawberry^41,42^. Another study suggested that *MYB86* promoted anthocyanin biosynthesis in red fruits by regulating genes involved in the flavonoids biosynthetic pathway^43^. Notably, the complex of *FaMYB9*/*FaMYB11*, *FabHLH3* and *FaTTG1* controls proanthocyanidin biosynthesis, competing with the anthocyanin biosynthetic pathway^44^. Here, we showed that *MYB86* was highly coexpressed with genes such as *UDP-glycosyltransferase* (Supplementary Table S4), implying that *MYB86* might be involved in a certain MYB/bHLH/WD complex for ABA-enhanced anthocyanin biosynthesis. Very recently, many WRKYs were characterized in the diploid strawberry genome^45,46^; however, their functions remain elusive. In octoploid strawberry, only *FaWRKY1* is reported with involvement in disease resistance^47^. Our results showed that *WRKY31*, *WRKY71* and particularly *WRKY65* responded positively to ABA, indicating that they might be critical for mediating the effect of ABA in fruit ripening. Additionally, our results indicated *KNOX* and *SPL18* as potential repressors of strawberry fruit ripening; however, more molecular evidence is required for this hypothesis.

The diverse roles of miRNA-directed gene regulation in plants have been widely characterized^9^. Our results revealed that miRNA expression changed in a different manner after ABA or NDGA application (Fig. 5), which might effectively influence the target genes involved in fruit development. For example, miR398b was downregulated in ABA-treated fruits in the present study (Fig. 5). Previously, in Arabidopsis, miR398 was negatively regulated under ABA stress^48^ but was induced by sucrose treatment^49^. Both ABA and sucrose play important roles during strawberry ripening^2,50^. MiR398 may be a convergent responder to ABA and sucrose and subsequently regulate its target genes encoding the copper superoxide dismutases, which are pivotal in alleviating oxidative stress^51^. MiR166d expression was downregulated by ABA during fruit development (Fig. 5) and might be involved in the regulation of the hormone balance between ABA and auxin during strawberry fruit ripening, because auxin and ABA are known to act antagonistically to control strawberry ripening^52^. Previously, csi-miR166d was thought to regulate the development of nonclimacteric citrus fruit by suppressing ATHB transcription factors, which are demonstrated to have a negative feedback module for regulating auxin signaling^21,53^. Therefore, ABA may inhibit auxin signaling through decreasing miR166d expression to switch the strawberry fruit from early developmental stages to the ripening phase. Moreover, miRNAs may also participate in the regulation of processes such as anthocyanin biosynthesis. In a previous study in Arabidopsis, stress-increased miR156 repressed the expression of its target SPL transcription factors, which promoted the expression of *dihydroflavonol-4-reductase* and elevated anthocyanin content^54^. We observed that miR156c was continuously induced by ABA (Fig. 5). As a consequence, this induction of miR156c might contribute to the suppression of *SPL18* in the coexpression network (Fig. 4), which subsequently might accelerate anthocyanin biosynthesis in ABA-treated fruits. In addition to known miRNAs, novel miRNAs or nonconserved miRNAs are also very important for fruit ripening^13^. Several novel miRNAs were identified that could be candidates for regulators controlling strawberry ripening. For example, ABA-induced Fa_novel23 may in turn inhibit the translation of the target *KFB* gene, resulting in the rapid accumulation of fruit pigments. Previously, *KFB* was demonstrated to be a negative regulator in flavonoid accumulation in *Cucumis melo* by shifting the metabolic flux toward the phenylpropanoid pathway^55^. Therefore, Fa_novel23 may be involved in controlling anthocyanin biosynthesis. As another example, Fa_novel11 may affect the mechanical and textural characteristics of fruit, because its target gene *PMEI* encodes a vital inhibitor for pectin methylesterases. Thus, the reduced expression of Fa_novel11 in NDGA-treated fruits might result in an increased level of *PMEI* transcripts; thereafter, the activity of pectin methylesterases was inhibited by PMEI and softening was then slowed. Our identification of known and new miRNAs and their target genes during fruit ripening establishes the foundation for future functional studies of miRNAs in fruit development.

A lack of significant differences in the expression of the target genes of miR164c, miR172b and miR396c suggested additional levels and modes of regulation. Fa_novel6 and *HERK1* was one notable miRNA−target mRNA pair*. HERK* encodes a receptor-like kinase, belonging to the *Catharanthus roseus* RLK1-Like family. HERK1 is one of the four members that have been implicated in regulating cell wall function in Arabidopsis^56^. Fa_novel6 likely modulated fruit size by modulating *HERK1* expression level during strawberry ripening. In Arabidopsis, the *herk1-the1* double mutant displayed an obvious dwarf phenotype. HERK1 and THE1 share redundant functions in promoting cell elongation^22^.

In conclusion, our study showed the regulatory function of ABA in different pathways for strawberry fruit ripening. Transcription factors belonging to WRKY and HSF families as well as miRNAs such as miR156 and Fa_novel 23 could be important regulators in ABA-induced strawberry receptacle ripening. In addition, we discovered that a novel miRNA, Fa_novel6, could degrade its target, *HERK1*, which is a receptor-like kinase in Arabidopsis with an activity in cell wall modification and cell elongation. Therefore, by regulating the expression of Fa_novel6, ABA may induce the expression level of *HERK1* and subsequently accelerate fruit enlargement.

## Materials and methods

### Plant material and treatments

Octoploid strawberry (*Fragaria ananassa* ‘Toyonoka’) plants were grown in greenhouses (60–80% relative humidity) under cycles of 14 h light at 25 °C followed by 10 h dark at 20 °C. Flowers were tagged during anthesis in the spring season of 2015. Two-week-old (post anthesis) fruits were treated with 100 μL of exogenous ABA (1 μM) or NDGA (an ABA biosynthesis blocker, 100 μM) solution. ABA or NDGA was first dissolved with a little ethanol (2% final) and then diluted with distilled water. Fruits treated with distilled water (2% ethanol) were used as the control (CK). The solutions were injected into each fruit receptacle core through the pedicel with a sterile 100 μL microsyringe as described by Jia et al.^2^. On the treatment day, after injection, fruit achenes were immediately removed using a scalpel, and the receptacles were immersed in liquid nitrogen and stored as CK0 (one replicate). Then, 20 fruits were sampled of each treatment following the same procedure 5 and 8 days after the treatment. Correspondingly, the sample names were set as CK5, ABA5, NDGA5, CK8, ABA8, and NDGA8. Two biological replicates (20 fruits for each replicate and treatment and time point) at an interval of >15 days were used for the sequencing analyses.

### Determination of fruit color, fruit size, and ABA content

Fruit surface color was determined by measuring four different locations around the equatorial region on each fruit using a Chroma meter (KONICA MINOLTA, CR-400, Japan). The results are presented as a* values in the L*a*b* color space. Fruit diameter and length were determined with a Vernier caliper. Ten fruits were used for the test. ABA content was determined as described previously^12^. Both exogenous and endogenous ABA were determined without separation.

### RNA extraction, library construction, and Illumina sequencing

Total RNA was isolated using RNAiso Plus (TaKaRa, Japan) following the manufacturer’s protocol. For each sample, RNA was extracted separately from ten randomly selected individual fruit receptacles. Before intrasample pooling, individual total RNA quantity and concentration were measured on a Bioanalyzer 2100 and with an RNA 6000 Nano LabChip Kit (Agilent, CA, USA) with RNA integrity number (RIN) > 8.0. The intrapooled sample was set as one biological replication, and two replications were prepared for the deep-sequencing analysis. After pooling, 14 RNA samples were prepared for cDNA library construction. Paired-end Illumina mRNA libraries were generated in accordance with the manufacturer’s instructions for RNA-seq sample preparation (Illumina Inc., San Diego, CA, USA). Afterward, the 14 libraries underwent sequencing on an Illumina HiSeq 4000 platform, and 150 bp paired-end reads were generated. To obtain a high alignment score, 100 bp reads were removed for analyses. The original data were deposited in the NCBI GEO database under access no. GSE85572.

### De novo assembly and bioinformatics analysis

Raw sequencing reads were filtered before de novo assembly to exclude low-quality reads, including adaptors, low-quality sequences (reads with >5% ambiguous bases) and reads with a Phred quality score (Q) ≤ 20 using an NGS QC toolkit. The remaining clean reads were assembled using the Trinity program^57^. Assembled unigenes were aligned against the reference genomes of both *Fragaria vesca*^58^ and *Fragaria ananassa*^59^. The top BLAST hit for each Trinity sequence was recorded from the genomes of both organisms along with the functional annotation for those hits. *Fragaria vesca* functional annotations were obtained from PLAZA (https://bioinformatics.psb.ugent.be/plaza/versions/plaza_v3_dicots/), and *Fragaria ananassa* annotations were obtained from the Strawberry GARDEN site (http://strawberry-garden.kazusa.or.jp/). Additionally, annotations from the Swiss-Prot database, Kyoto Encyclopedia of Genes and Genomes (KEGG), euKaryotic Ortholog Groups (KOG), and Pfam protein databases using the BLASTx algorithm with an E-value cutoff of 10^−10^ are also provided in Supplemental Table S1. Gene expression levels in each sample were normalized by applying the read counts per kilobase of exon model per million reads (RPKM) method^60^. Differentially expressed genes were analyzed with DESeq in R between sample groups (*p*_adj_ < 0.05). Quantitative reverse transcription PCR (qRT-PCR) was applied to validate the deep-sequencing data. *FaACTIN* was used as the reference gene, and the primers used for qRT-PCR are listed in Supplementary Table S8.

### Small RNA and degradome library construction, sequencing and data processing

Small RNA libraries of the 14 samples were prepared for sRNA analysis according to the protocol of a TruSeq Small RNA Sample Prep Kit (Illumina, San Diego, USA). Single end sequencing (50 bp) was performed on an Illumina HiSeq 2000 at LC-BIO (Hangzhou, China) following the vendor’s protocol. The sRNA sequencing data were processed as described previously^11^. The analysis pipeline of miRNA sequencing data is presented in Supplemental Fig. S2. The miRbase v 21.0 was applied to identify known miRNAs and predict novel miRNAs in strawberries. The differential expression levels of miRNAs, based on normalized deep-sequencing counts, were analyzed using DESeq in R. Additionally, degradome cDNA libraries were constructed from the same fruit samples used for transcriptome and miRNA sequencing by following the procedures previously described by German et al.^61^. The CleaveLand 3.0 software package and the ACGT301-DGE v1.0 program (LC Sciences, Houston, TX, USA) were used to analyze the degradome sequencing data^62^. The expression of identified miRNAs was validated by qRT-PCR with stem-loop primers. All reactions were performed in triplicate with 5.8S rRNA as the internal reference. The primers are listed in Supplementary Table S8.

### Bioinformatic analyses

The dendrogram in Fig. 1c was created by the function *cor ()* in R with default settings, which reflected the degree of gene expression variance. In Fig. 2, the 4164 differentially expressed genes were divided into nine clusters by K-means clustering on the website http://www.bioinf.ebc.ee/EP/EP/EPCLUST/ with Euclidean distance. The clustering process was based on expression fold change in the fruit samples of CK0, CK5 and CK8. For Fig. 3, the heat map was prepared in MeV4.9 by importing a relative value that was calculated according to the method reported by Yu et al.^63^. Identification of TFs was performed with blasting to the PlantTFDB database at http://planttfdb.cbi.pku.edu.cn. The networks in Fig. 4 were visualized using Cytoscape _v.3.0.0 with a cutoff >0.9 of the correlation rate between the expression of TFs and differentially expressed genes. The hierarchical clustering in Fig. 5 was created with the application of average linkage clustering and Pearson correlation within MeV4.9. The secondary structure of miRNAs was analyzed on an RNAfold Webserver (http://rna.tbi.univie.ac.at/cgi-bin/RNAWebSuite/RNAfold.cgi).

## Electronic supplementary material

Table S1

Table S2

Table S3

Table S4

Table S5

Table S6

Table S7

Table S8

fig s1

fig s2

Supplementary data
