# Supplementary figures and images for: Integrated analysis of high-throughput sequencing data shows abscisic acid-responsive genes and miRNAs in strawberry receptacle fruit ripening
Source: Hortic Res. 2019 Feb 1;6:26. doi: 10.1038/s41438-018-0100-8 (PMC6355886; doi:10.1038/s41438-018-0100-8)

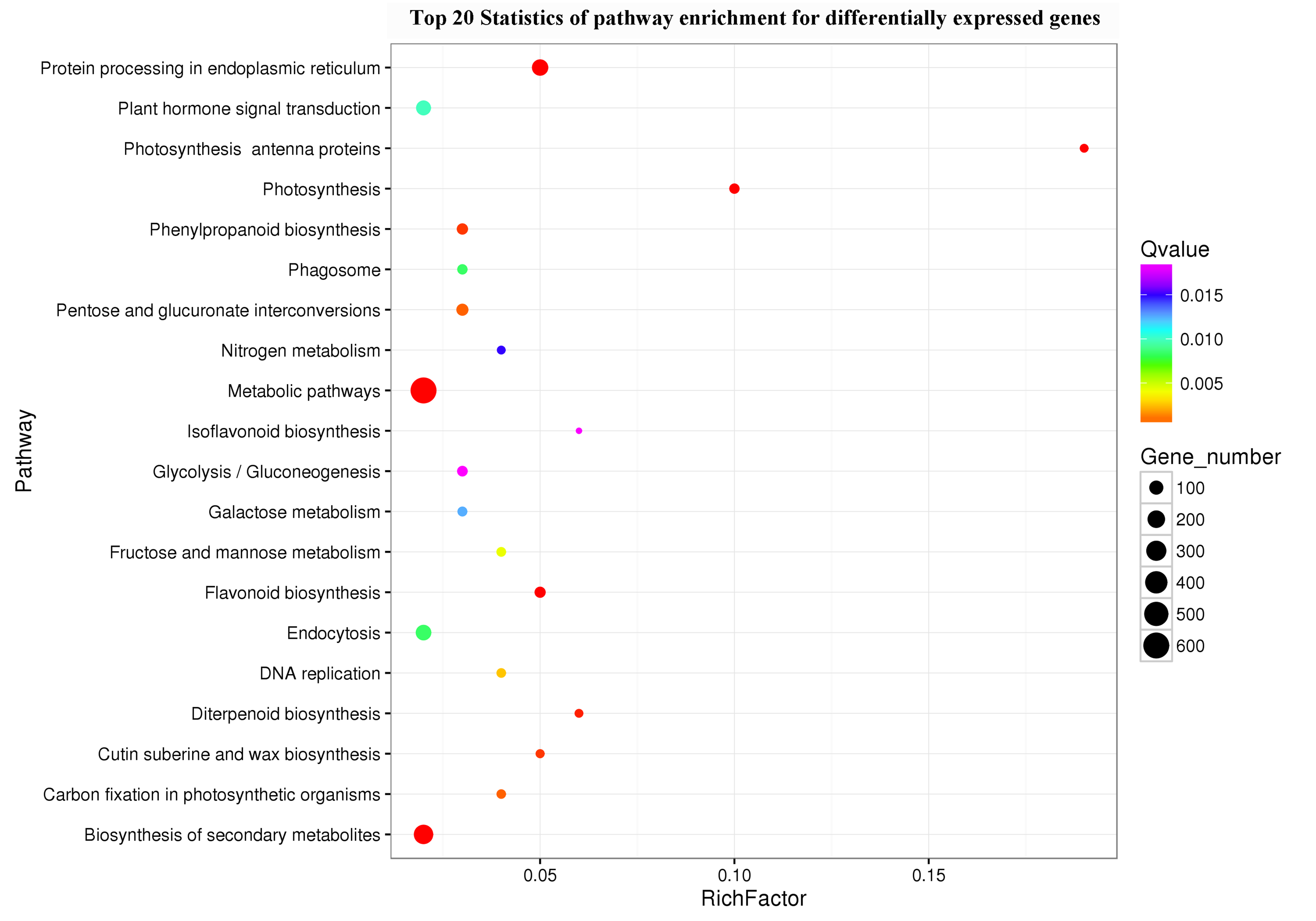

Supplement: Supplementary file 9 — fig s1 [file 41438_2018_100_MOESM9_ESM.tif]

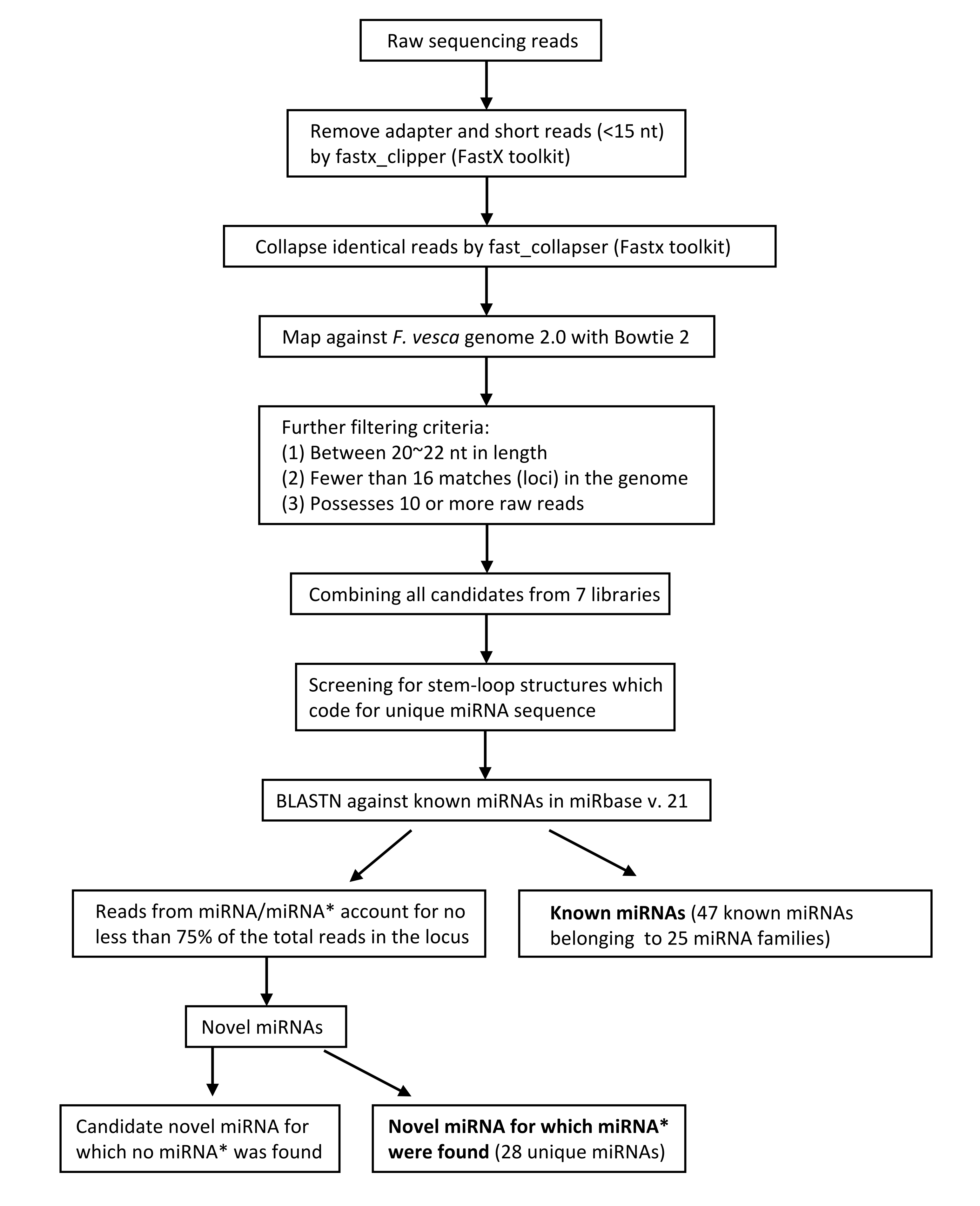

Supplement: Supplementary file 10 — fig s2 [file 41438_2018_100_MOESM10_ESM.tif]
