# Supplementary material for: Integrated analysis of high-throughput sequencing data shows abscisic acid-responsive genes and miRNAs in strawberry receptacle fruit ripening
Source: Hortic Res. 2019 Feb 1;6:26. doi: 10.1038/s41438-018-0100-8 (PMC6355886; doi:10.1038/s41438-018-0100-8)
Supplement: Supplementary file 11 — Supplementary data [file 41438_2018_100_MOESM11_ESM.docx]

**Supplementary data**
The online version of this article contains supplementary materials.

Fig. S1. KEGG pathway analysis of the 4164 differentially expressed genes. The x-axis shows the enriched factor. The y-axis indicates the KEGG pathway. Gene number assigned to a specific pathway is presented as circle size.

Fig. S2. The pipeline of miRNA data analysis.

Table S1. All unigenes identified and annotations by blasting against the genomes of the two species *Fragaria ananassa* and *Fragaria vesca*.

Table S2. 4164 significantly differentially expressed genes were clustered into 9 categories according to their expression in control fruits.

Table S3. ABA-regulated genes involved in plant hormone signal transduction and biosynthesis of secondary metabolites pathways.

Table S4. Transcription factors and genes regulated by ABA.

Table S5. Statistics of miRNA sequencing data.

Table S6. All known and novel miRNAs identified during strawberry fruit ripening.

Table S7. Statistics of degradome sequencing data and miRNA-targeted genes detected by degradome sequencing.

Table S8. qRT-PCR validation of the transcriptome and miRNA sequencing data. CK, ABA and NDGA represent the control and abscisic acid and nordihydroguaiaretic acid-treated fruits, respectively. The number in the sample name indicates days after the treatment.
